# Evaluation of Glutathione Peroxidase 4 role in Preeclampsia

**DOI:** 10.1038/srep33300

**Published:** 2016-09-19

**Authors:** Xinguo Peng, Yan Lin, Jinling Li, Mengchun Liu, Jingli Wang, Xueying Li, Jingjing Liu, Xuewen Jia, Zhongcui Jing, Zuzhou Huang, Kaiqiu Chu, Shiguo Liu

**Affiliations:** 1 Clinical Laboratory, Binzhou Medical University Hospital, Binzhou, China; 2 Clinical Laboratory, the Affiliated Hospital of Qingdao University, Qingdao, China; 3 Department of Intensive Care Unit, the Affiliated Hospital of Qingdao University, Qingdao, China; 4 Prenatal Diagnosis Center, the Affiliated Hospital of Qingdao University, Qingdao, China; 5 Department of Histology and Embryology, Qingdao University Medical College, Qingdao, China; 6 Department of Blood Transfusion, the Affiliated Hospital of Qingdao University, Qingdao, China; 7 Department of cardiovascular, the Affiliated Hospital of Qingdao University, Qingdao, China; 8 Departmet of Haematology, Qingdao University Medical College, Qingdao, China

## Abstract

Preeclampsia (PE) is a pregnancy-specific syndrome that may be lifethreatening to pregnancies and fetus. Glutathione Peroxidase 4 (GPx4) is a powerful antioxidant enzyme that can provide protection from oxidative stress damage which plays a pivotal role in the pathology of PE. Therefore, this study aims to investigate the association between *Gpx4* polymorphisms and the susceptibility to PE in Chinese Han women. TaqMan allelic discrimination real-time PCR was used to perform the genotyping of rs713041 and rs4807542 in 1008 PE patients and 1386 normotensive pregnancies. Obviously statistical difference of genotypic and allelic frequencies were found of rs713041 in *GPx4* between PE patients and controls and the C allele has the higher risk for pathogenesis of PE (χ^2^ = 12.292, P = 0.002 by genotype; χ^2^ = 11.035, P = 0.001, OR = 1.216, 95% CI 1.084–1.365 by allele). Additionally, when subdividing these samples into CC + CT and TT groups, we found a significant difference between the two groups (χ^2^ = 11.241, P = 0.001, OR = 1.417, 95% CI 1.155–1.738). Furthermore, the genotype of rs713041 was found to be associated with the mild, severe and early-onset PE. Our results suggest that rs713041 in *GPx4* may play a key role in the pathogenesis of PE.

Preeclampsia (PE) is an unpredictable and potentially dangerous clinical complication that occurs during the second half of pregnancy, characterized by the new onset hypertension and proteinuria after 20^th^ week of gestation^1^. It is a leading cause of perinatal morbidity and mortality worldwide for which is often accompanied with multi-organ disorders such as liver rupture, stroke, pulmonary oedema, or kidney failure, which can all be lethal without effective treatment except for terminating pregnancy^2^,^3^. Although extensive researches have been made for many years, the etiology and pathophysiology of this disorder remains elusive. Several factors may be associated with the development of PE, such as oxidative stress, inflammation and hereditary variants^4^,^5^.

Strong evidence exists that oxidative stress plays a pivotal role in the pathology of PE^6^,^7^,^8^. Oxidative stress is a state in which the balance between reactive oxygen species (ROS) and the antioxidant host defenses is disturbed and ROS prevails over the antioxidant host defenses^9^,^10^,^11^. ROS include free radical species which borne unpaired electrons and their non-radical intermediates, such as superoxide (O_2_^−^), hydrogen peroxide(H_2_O_2_), hydroxylradical (^·^OH). The anti-oxidants, e.g. glutathione peroxidase (GPx), superoxide dismutase(SOD), catalase, vitamins C and E, and b-carotene played a significant role in scavenging free radicals^12^. During normal gestation, ROS and the anti-oxidants are in the balanced status. However, when ROS increase or the anti-oxidants decrease, thnificante pregnancies will be in a oxidative state which may involve in the etiopathogenesis of PE^13^.

GPx4 is a powerful antioxidant enzyme that is capable of metabolizing H_2_O_2_ and lipid hydroperoxides to water or the corresponding unreactive alcohols which can provide protection from oxidative damage^14^. A cohort study revealed that *GPx4* mRNA expression decreased in PE patients compared to their respective control group. Mistry and his colleagues reported that GPx4 was in a lower level in PE than controls^15^,^16^. Therefore, decreased expression of GPx4 may be associated with the development of PE. In addition, GPx4 is also reported to be involved in other functions such as male fertility and apoptosis^17^. Moreover, epidemiological research revealed that genetic factors appeared to have an important role in the pathology of PE and significant associations between PE and candidate genes variants that is involved in oxidative stress have been identified^4^,^5^,^18^. Additionally, two tag SNPs (rs713041 and rs4807542) in G*Px4* whose product belonged to antioxidant enzymes were reported to have an influence on its expression possiblly^19^. Therefore, in this case-control study, we aim to explore the association between the polymorphisms of G*Px4* and the susceptibility to PE in Chinese Han women.

## Results

### Demographic and clinical characteristics

Table 1 shows the comparison in demographic and clinical characteristics between cases and controls. The age of cases and controls was 30.45 ± 5.06 and 30.25 ± 3.91 years old respectively, which was matched between both groups (P = 0.302). We have not observed significant differences in times of gravidity (P = 0.712), number of abortions (P = 0.179) and age of menarche (P = 0.833) between cases and controls. However, compared to controls, PE patients had earlier admitted and delivered weeks. higher blood pressure and lower birth weight of offspring (P < 0.001).

### Genetic analysis

The participants of control group in our study were in accordance with HWE for both SNPs and had a group representative (for rs713041, χ^2^ = 2.571, P = 0.109; for rs4807542, χ^2^ = 0.046, P = 0.830).

The genotypic and allelic frequencies of rs713041 and rs4807542 in cases and controls were summarized in Table 2. For rs713041, the genotypic and allelic distributions between two groups were statistically different (χ^2^ = 12.292, P = 0.002 by genotype; χ^2^ = 11.035, P = 0.001,OR = 1.216, 95% CI 1.084–1.365 by allele). When subdividing these samples into CC and CT + TT groups, no obvious difference between cases and controls was found (χ^2^ = 4.458, P = 0.035, OR = 1.208, 95% CI 1.014–1.439). Then, when subdividing these samples into CC + CT and TT groups, we found a significant difference between cases and controls (χ^2^ = 11.241, P = 0.001, OR = 1.417, 95% CI 1.155–1.738). The C allele of rs713041 was related to the development of PE (χ^2^ = 11.035, P = 0.001, OR = 1.216, 95% CI 1.084–1.365). Nevertheless, no statistical differences were observed in rs4807542 between the two groups in terms of genotypic frequencies (χ^2^ = 2.037, P = 0.361), nor for allelic frequencies (χ^2^ = 0.346, P = 0.556, OR = 0.947, 95% CI 0.791–1.134).

We divided PE patients into mild and severe PE groups according to guidelines from the American College of Obstetricians and Gynecologists in order to further explore the association between the variants of both SNPs and PE^20^. The genotypic and allelic frequencies of rs713041 and rs4807542 in mild or severe PE were compared with those in normotensive pregnancies respectively. In Table 3 statistical differences between mild/severe PE patients and normotensive controls in both genotypic distributions and allelic frequencies of rs713041 were observed (mild PE vs. control: χ^2^ = 10.716, P = 0.005 by genotype; χ^2^ = 10.981, P = 0.001, OR = 1.497, 95% CI 1.178–1.903 by allele; severe PE vs. control: χ^2^ = 8.572, P = 0.014 by genotype; χ^2^ = 6.509, P = 0.011, OR = 1.171, 95% CI 1.037–1.323 by allele). However, the mild/severe PE patients did not differ significantly from the controls in either genotypic distributions or allelic frequencies of rs4807542 (mild PE vs. control: χ^2^ = 0.275, P = 0.872 by genotype; χ^2^ = 0.001, P = 0.978, OR = 0.995, 95% CI 0.693–1.429 by allele; severe PE vs. control: χ^2^ = 2.077, P = 0.354 by genotype; χ^2^ = 0.427, P = 0.513,OR = 0.939, 95% CI 0.776–1.135 by allele).

We defined early-onset PE patients as those diagnosed before the 34^th^ week of gestation, known to be more severely affected than those with later-onset PE^21^. As shown in Table 4, we identified significant differences in both genotype and allelic frequencies of rs713041 between early-onset PE patients and controls while no statistical siginificances were found between patients with later-onset PE and normotensive pregnancies (early-onset PE vs. control: χ^2^ = 10.896, P = 0.004 by genotype; χ^2^ = 10.825, P = 0.001, OR = 1.273, 95% CI 1.102–1.470 by allele; later-onset PE vs. control: χ^2^ = 5.175, P = 0.075 by genotype; χ^2^ = 3.841, P = 0.052,OR = 1.159, 95% CI 1.000–1.342 by allele). For rs4807542, there were no statistical diffrences in genotypic distributions and allelic frequencies between early/late-onset PE and control groups (early-onset PE vs. control: χ^2^ = 2.678, P = 0.262 by genotype; χ^2^ = 1.126, P = 0.289, OR = 1.146, 95% CI 0.891–1.475 by allele; later-onset PE vs. control: χ^2^ = 2.917, P = 0.233 by genotype; χ^2^ = 2.196, P = 0.138, OR = 0.853, 95% CI 0.691–1.053 by allele).

### Analysis of genotype-phenotype relationship

Table 5 demonstrated the results of a detailed genotype-phenotype analysis of rs713041 which involved clinical characteristics and laboratory examination. When PE patients were divided into CC, CT and TT groups, no significant differences in the parameters between the three groups were found, including systolic pressure, diastolic pressure, birth weight of offspring, white blood cell count (WBC), neutrophil count, red blood cell count (RBC), haemoglobin (Hb), platelet (PLT), prothrombin time (PT), activated partial thromboplastin time (APTT), alanine transaminase (ALT), aspartate transaminase (AST), urea nitrogen, creatinine (P > 0.05). Moreover, there were no statistical siginificances either when PE patients were fractionized into CC/CT + TT groups and CC + CT/ TT groups (P > 0.05).

## Discussion

PE is a pregnancy-specific syndrome and may be accompanied with severe complications when left untreated, which will threaten the health of mothers and fetus^22^. Although the etiology of PE is unclear, some causes have been reported that may have a possible role in the development of the disorder. A causal relationship between oxidative stress and the pathophysiology of PE was demonstrated to exist^23^,^24^. Oxidative stress arises from an imbalance between the generation of ROS and the antioxidants that prevent harmful effects of ROS. Various molecules, including lipids, proteins, and DNA. can be affected and damaged by this disparity^9^.

GPx4 is an intracellular antioxidant selenoprotein which can provide protection from oxidative damage. It is a monomer that consists of a cytosolic (cGPx4), a mitochondrial (mGPx4) and sperm nuclear GPx4 (snGPx4) isoforms. cGPx4 exists in cells ubiquitously; mGPx4, as well as snGPx4, mainly exists in testis while only trace amounts are expressed in other tissues^17^,^25^. It plays a pivotal role in prohibiting the development of oxidative stress by reducing H_2_O_2_ and lipid hydroperoxides to water or the corresponding alcohols. Furthermore, it can directly reduce bound phospholipid hydroperoxides within membranes and lipoproteins, which is the only enzymatic antioxidant known to have this unique function of all the GPxs^26^. Because of the significant function of GPx4 to counteract oxidative stress, decrease in GPx4 level may be associated with the pathogenesis of PE, in which oxidative stress plays a key role. Evidences also indicated that the GPx4 mRNA and protein level were lower in PE patients than those in their normotensive counterparts^15^,^16^. In addition, GPx4 is also reported to be involved in other cell functions such as male fertility, apoptosis and modulating inflammation through regulating leukotriene biosynthesis and cytokine signaling pathways^17^,^27^.

*GPx4*, located in chr19p13.3, comprised by 7 exons and several introns in human. Regina Brigelius-Flohé found that it was lethal to the mice when *gpx4* was knocked out entirely from the whole genome^17^. Variants in *GPx4* could influence its antioxidant capacity and other functions^28^. Two candidate SNPs (rs713041 and rs4807542) in *GPx4* were chosen as the tag SNPs. The polymorphism of rs713041, located in the 3′UTR which is near the Sec insertion sequence element at position 718, could modulate the synthesis of *GPx4* through changing the affinity of the Sec insertion machinery for its Sec insertion sequence element and protein that combines with the 3′UTR^19^. Moreover, lipid metabolism and several cancers are reported to be related to this polymorphism^29^,^30^,^31^,^32^. Both SNPs are seated in the exons and might influence the gene expression^19^. As these two SNPs might affect the expression of *GPx4* and the antioxidant capacity of its product which is pivotal in the pathogenesis and development of PE, we performed this case–control study to explore the relationship between polymorphism of *GPx4* and PE.

In our study, we observed significant differences in genotypic and allelic frequencies of rs713041 between PE patients and their normotensive counterparts, which can demonstrate an association between G*Px4* rs713041 polymorphism and PE. Furthermore, for rs713041, the C allele was higher in PE patients than that in normal controls and may be the risk allele for the development of PE by calculating of the value of OR. When cases were divided into CC + CT/TT groups, we found significant differences with OR1.417, through which we can conclude that patients with CC or CT genotype were more inclined to develop PE than patients with TT genotype. However, we failed to identify an association between the genotype or allelic frequencies of rs4807542 and PE.

In addition, when subdividing cases into mild/severe or early-onset/late-onset groups, the genotypic distributions and allelic frequencies of rs713041 were associated with mild PE, severe PE and early-onset PE with OR1.497, OR1.171, OR1.273 by allele respectively. However, for rs4807542 no statistically significant differences were demonstrated between healthy pregnancies and patients with PE in all these subgroups. In conclusion, our results suggest that the polymorphism of rs713041 in *GPx4* may play a pivotal role in the pathogenesis and progression of PE.

To our knowledge, this was the first time to investigate the polymorphisms of *GPx4* in patients with PE. However, previous studies had explored the association between genetic polymorphisms in other oxidative stress related genes and PE. Ji Hyae Limsuch *et al*. indicated that the variant genotype of the *COMT* (MetMet) was associated with an increased risk of PE^33^; Ebru *et al*. demonstrated that the −463G/A polymorphism of *MPO* could be an intriguing susceptibility factor of PE in Turkish population^34^; and Lucia and his colleagues proposed that polymorphisms of rs4880 in *SOD2* was significantly associated with PE^35^. In addition, it was reported that rs713041 in *GPx4* was associated with idiopathic recurrent miscarriage and it could be regarded as a predictor of cerebral stroke in Russian patients with essential hypertension, while Xiao *et al*. stated that both SNPs were related to the development of Kashin-Beck disease in the Chinese population^19^,^36^,^37^.

A limitation of our study is that all participants were ethnic Han Chinese coming from Shandong province. As regional and racial differences are probably to influence the results, our observations could not represent other human races. Furthermore, we did not explore the relationship between PE and environmental factors such as diet, smoking and stress which may participate in the pathogenesis and development of PE. In addition, one or even several genetic variants might not influence gene expression for that PE is a multifactorial hereditary disease but we only investigated two SNPs of *GPx4*. The relationship between rs713041 and the susceptibility to PE may be direct association meaning that the site rs713041 has function such as regulating gene transcription, influencing gene expression and so on. However, the association may also be indirect meaning that the site rs713041 has linkage disequilibrium with other adjacent functional sites, which just is a linkage marker without function itself. Therefore, functional experiments measuring GPx4 expression and activity need to be conducted to strengthen our conclusions in the future. Despite some limitations, our study suggested that *GPx4* may involve in the development of PE in Chinese Han pregnancies. Larger-scale studies involving candidate genes and variant SNPs are necessary to be conducted in different races and regions with functional and environmental analyses to validate our findings and further explore the pathogenesis of PE.

## Methods

### Subjects

We recruited 1008 diagnosed PE patients as the case group and 1386 age-matched normotensive pregnencies as the control group. All the subjects are Chinese Han women that come from Binzhou Medical University Hospital, the Affiliated Hospital of Qingdao University, Linyi People’s Hospital, Liaocheng People’s Hospital, the Maternal and Child Health Care of Zaozhuang, Yantai Yuhuangding Hospital and Yantaishan Hospital between January 2013 and November 2015. The research staffs filled out the questionnaire which contains maternal age, gestational weeks of addmition and delivery, blood pressure, pregnancy and delivery history, clinical symptoms, and results of laboratory examinations. All PE patients and normal controls signed the informed consent. Our study was approved by the ethics committee of the Affiliated Hospital of Qingdao University and the methods were carried out in accordance with the relevant guidelines.

PE was defined as onset of hypertension (≥140/90 mmHg) with detectable urinary protein (≥0.3 g/24 h, or ≥1 + by dipstick) after 20 weeks of gestation which may be accompanied by headache, blurred vision and upper abdominal discomfort. Both PE and control pregnant women are age matched and have no previous history of PE and a systemic disease such as chronic hypertension, heart disease, diabetes mellitus, thyroid function disorder, kidney disorders, hepatic diseases excluding from artificial insemination, twin or multiple pregnancy, and macrosomia in the present gestation.

### Genetic studies

DNA was isolated from peripheral blood lymphocytes using a Qiagen DNA extraction kit (Qiagen, Hilden, Germany). The TaqMan allelic discrimination real-time PCR was used to genotype the polymorphisms of rs713041 and rs4807542 in *GPx4*. We used the Taqman probes and primers which were designed by Applied Biosystems of Life Technologies (New York, USA). The primers of rs713041 were 5′-CCGCCCGAGCCCCTGCCCACGCCCT-3′ (forward) and 5-GGAGCCTTCCACCGGCACTCATGAC-3′ (reverse). The primers of rs4807542 were 5′-GCCGCCTTTGCCGCCTACTGAAGCC-3′ (forward) and 5′-GCGCTGCTCTGTGGGGCTCTGGCCG-3′ (reverse). The polymerase chain reaction (PCR) system which was 25 μl totally consisted of 1.25 μl 20 × SNP Genotyping Assay, 12.5 μl 2 × PCR Master Mix, and 11.25 μl DNA and DNase-free water. C1000TM thermal cycler system was used to conduct the Amplifications with the following conditions: 95 °C for 3 min, followed by 45cycles at 95 °C for 15 sec and 60 °C for 1 min. The fluorescent signals from VIC/FAM-labeled probes were detected each cycle. Bio-Rad CFX manager 3.0 software was used to conduct the genotyping.

### Statistical analysis

We used statistical software package SPSS 21.0(SPSS Inc., Chicago, IL, USA) to perform all analyses. Hardy—Weinberg equilibrium (HWE) was assessed in the controls by chi-square test. Differences between cases and controls in demographic and clinical characteristics were compared using Student’s t-test. The genotype-phenotype analysis was conducted by an analysis of variance (ANOVA). Statistical significance was set at p < 0.05 (two-sided). We used Pearson’s χ^2^ test (if expected values were below 5, Fisher’s exact test was used) to compare differences in allelic and genotypic distributions between two groups and a level of P < 0.025 (two-sided) was considered statistically significant when Bonferroni’s correction was made. Odds ratios (ORs) and 95% confidence intervals (CIs) were used to show the relative risk degree.

## Additional Information

**How to cite this article**: Peng, X. *et al*. Evaluation of Glutathione Peroxidase 4 role in Preeclampsia. *Sci. Rep.* **6**, 33300; doi: 10.1038/srep33300 (2016).

## Figures and Tables

**Table 1:** The demographic and clinical characteristics of PE and control groups.

Characteristics	PE	Control	t	p-value
Maternal age (years)	30.45 ± 5.06	30.25 ± 3.91	1.033	0.302
Age of menarche (years)	14.01 ± 1.18	14.00 ± 1.25	0.21	0.833
Gestational age at admission (weeks)	35.24 ± 3.53	39.20 ± 1.38	−33.691	<0.001
Gestational age at delivery (weeks)	35.80 ± 3.15	39.43 ± 1.15	−33.676	<0.001
Times of gravidity	2.25 ± 1.27	2.24 ± 1.16	0.369	0.712
Number of abortion	1.48 ± 0.67	1.42 ± 0.72	1.344	0.179
Systolic blood pressure (mmHg)	161.10 ± 18.68	114.71 ± 9.88	71.758	<0.001
Diastolic blood pressure (mmHg)	104.69 ± 13.69	73.44 ± 7.83	65.083	<0.001
Birth weight of offspring (g)	2505.91 ± 937.29	3405.94 ± 357.36	−27.698	<0.001

**Table 2:** The comparison of genotypic and allelic frequencies of both SNPs between PE and controls.

Group	N	rs713041							rs4807542				
		CC	CT	TT	CC/CT + TT	CC + CT/TT	C	T	AA	AG	GG	A	G
PE	1008	333	497	178			1163	853	19	188	801	226	1790
Control	1386	402	661	323			1465	1307	20	286	1080	326	2446
χ^2^		12.292			4.458	11.241	11.035		2.037			0.346	
p-value		0.002			0.035	0.001	0.001		0.361			0.556	
OR					1.208	1.417	1.216					0.947	
95% CI					1.014–1.439	1.155–1.738	1.084–1.365					0.791–1.134	

**Table 3:** The comparison of genotypic and allelic frequencies of both SNPs between mild/severe PE and control groups.

Group	N	rs713041					rs4807542				
		CC	CT	TT	C	T	AA	AG	GG	A	G
Mild PE	158	64	70	24	198	118	3	31	124	37	279
Control	1386	402	661	323	1465	1307	20	286	1080	326	2446
χ^2^		10.716			10.981		0.275			0.001	
p-value		0.005			0.001		0.872			0.978	
OR					1.497					0.995	
95% CI					1.178–1.903					0.693–1.429	
Severe PE	850	269	427	154			16	157	677	189	1511
Control	1386	402	661	323			20	286	1080	326	2446
χ^2^		8.572			6.509		2.077			0.427	
p-value		0.014			0.011		0.354			0.513	
OR					1.171					0.939	
95% CI					1.037–1.323					0.776–1.135	

**Table 4:** The comparison of genotypic and allelic frequencies of both SNPs between early/late-onset PE and control groups.

Group	N	rs713041					rs4807542				
		CC	CT	TT	C	T	AA	AG	GG	A	G
Early-onset PE	523	181	253	89	615	431	12	105	406	129	917
Control	1386	402	661	323	1465	1307	20	286	1080	326	2446
χ^2^		10.896			10.825		1.704			0.237	
p-value		0.004			0.001		0.427			0.615	
OR					1.273					1.056	
95% CI					1.102–1.470					0.849–1.312	
Late-onset PE	485	152	244	89	548	422	7	83	395	97	873
Control	1386	402	661	323	1465	1307	20	286	1080	326	2446
χ^2^		5.175			3.841		2.824			2.221	
p-value		0.075			0.052		0.244			0.136	
OR					1.159					0.834	
95% CI					1.000–1.342					0.656–1.059	

**Table 5:** Associations between genotypes of rs713041 and characteriscs among PE patients.

	①CC	②CT	③TT	①vs②vs③	①vs②	①vs③	②vs③	①vs② + ③	① + ②vs③
	Mean ± SD	Mean ± SD	Mean ± SD	p-value	p-value	p-value	p-value	p-value	p-value
Systolic blood pressure (mmHg)	162.06 ± 20.09	160.37 ± 17.91	161.33 ± 18.04	0.438	0.203	0.672	0.56	0.252	0.644
Diastolic blood pressure (mmHg)	105.05 ± 14.51	104.06 ± 13.46	105.81 ± 12.69	0.289	0.308	0.545	0.142	0.568	0.244
Birth weight of offspring (g)	2498.36 ± 944.44	2481.73 ± 937.38	2587.12 ± 924.74	0.465	0.811	0.33	0.22	0.863	0.348
RBC (×10^12^/L)	4.00 ± 0.65	4.05 ± 0.58	4.03 ± 0.52	0.549	0.275	0.559	0.792	0.287	0.83
Hb (g/L)	113.12 ± 17.76	115.67 ± 20.11	114.60 ± 17.54	0.164	0.057	0.399	0.518	0.074	0.624
WBC (×10^9^/L)	9.60 ± 3.02	9.65 ± 2.85	9.49 ± 2.70	0.811	0.789	0.687	0.519	0.951	0.611
neutrophil (×10^9^/L)	7.21 ± 2.76	7.18 ± 2.68	7.07 ± 2.44	0.845	0.87	0.569	0.636	0.737	0.709
PLT (×10^9^/L)	203.22 ± 69.88	207.16 ± 69.83	196.85 ± 72.23	0.237	0.429	0.329	0.094	0.797	0.205
PT (s)	10.07 ± 1.66	10.29 ± 1.71	10.05 ± 1.62	0.124	0.084	0.861	0.111	0.202	0.225
APTT (s)	31.02 ± 3.86	30.83 ± 3.79	30.70 ± 4.03	0.65	0.501	0.381	0.7	0.537	0.546
ALT (IU/L)	26.09 ± 43.32	26.30 ± 48.20	22.98 ± 32.65	0.68	0.945	0.454	0.395	0.825	0.499
AST (IU/L)	29.28 ± 35.96	30.55 ± 39.32	29.32 ± 36.70	0.87	0.635	0.991	0.711	0.708	0.779
urea nitrogen (mmol/L)	4.86 ± 2.15	4.90 ± 3.83	4.74 ± 1.87	0.846	0.848	0.689	0.563	0.451	0.96
creatinine (umol/L)	68.05 ± 33.21	66.59 ± 33.51	67.20 ± 23.46	0.814	0.521	0.774	0.83	0.545	0.861

## References

[b1] Hansson S. R., Naav A. & Erlandsson L. Oxidative stress in preeclampsia and the role of free fetal hemoglobin. Frontiers in physiology 5, 516, 10.3389/fphys.2014.00516 (2014). 25628568 PMC4292435

[b2] Steegers E. A., von Dadelszen P., Duvekot J. J. & Pijnenborg R. Pre-eclampsia. Lancet 376, 631–644, 10.1016/S0140-6736(10)60279-6 (2010). 20598363

[b3] Souza J. P. . Moving beyond essential interventions for reduction of maternal mortality (the WHO Multicountry Survey on Maternal and Newborn Health): a cross-sectional study. Lancet 381, 1747–1755, 10.1016/S0140-6736(13)60686-8 (2013). 23683641

[b4] Rana S., Karumanchi S. A. & Lindheimer M. D. Angiogenic factors in diagnosis, management, and research in preeclampsia. Hypertension 63, 198–202, 10.1161/HYPERTENSIONAHA.113.02293 (2014). 24166749 PMC3947285

[b5] Jebbink J. . Molecular genetics of preeclampsia and HELLP syndrome - a review. Biochimica et biophysica acta 1822, 1960–1969, 10.1016/j.bbadis.2012.08.004 (2012). 22917566

[b6] Redman C. W. & Sargent I. L. Placental debris, oxidative stress and pre-eclampsia. Placenta 21, 597–602, 10.1053/plac.2000.0560 (2000). 10985960

[b7] Hubel C. A. Oxidative stress in the pathogenesis of preeclampsia. Proceedings of the Society for Experimental Biology and Medicine. Society for Experimental Biology and Medicine 222, 222–235 (1999). 10.1177/153537029922200305 10601881

[b8] Burton G. J. & Jauniaux E. Placental oxidative stress: from miscarriage to preeclampsia. Journal of the Society for Gynecologic Investigation 11, 342–352, 10.1016/j.jsgi.2004.03.003 (2004). 15350246

[b9] Kalyanaraman B. Teaching the basics of redox biology to medical and graduate students: Oxidants, antioxidants and disease mechanisms. Redox biology 1, 244–257, 10.1016/j.redox.2013.01.014 (2013). 24024158 PMC3757692

[b10] Lappas M., Mitton A. & Permezel M. In response to oxidative stress, the expression of inflammatory cytokines and antioxidant enzymes are impaired in placenta, but not adipose tissue, of women with gestational diabetes. The Journal of endocrinology 204, 75–84, 10.1677/JOE-09-0321 (2010). 19833719

[b11] Matsubara K., Matsubara Y., Hyodo S., Katayama T. & Ito M. Role of nitric oxide and reactive oxygen species in the pathogenesis of preeclampsia. The journal of obstetrics and gynaecology research 36, 239–247, 10.1111/j.1447-0756.2009.01128.x (2010). 20492372

[b12] Cindrova-Davies T. Gabor Than Award Lecture 2008: pre-eclampsia - from placental oxidative stress to maternal endothelial dysfunction. Placenta 30 Suppl A, S55–S65, 10.1016/j.placenta.2008.11.020 (2009). 19118896

[b13] Sanchez-Aranguren L. C., Prada C. E., Riano-Medina C. E. & Lopez M. Endothelial dysfunction and preeclampsia: role of oxidative stress. Frontiers in physiology 5, 372, 10.3389/fphys.2014.00372 (2014). 25346691 PMC4193194

[b14] Brigelius-Flohe R. Glutathione peroxidases and redox-regulated transcription factors. Biological chemistry 387, 1329–1335, 10.1515/BC.2006.166 (2006). 17081103

[b15] Roland-Zejly L., Moisan V., St-Pierre I. & Bilodeau J. F. Altered placental glutathione peroxidase mRNA expression in preeclampsia according to the presence or absence of labor. Placenta 32, 161–167, 10.1016/j.placenta.2010.11.005 (2011). 21145108

[b16] Mistry H. D. . Differential expression and distribution of placental glutathione peroxidases 1, 3 and 4 in normal and preeclamptic pregnancy. Placenta 31, 401–408, 10.1016/j.placenta.2010.02.011 (2010). 20303587

[b17] Brigelius-Flohe R. & Maiorino M. Glutathione peroxidases. Biochimica et biophysica acta 1830, 3289–3303, 10.1016/j.bbagen.2012.11.020 (2013). 23201771

[b18] Gurdol F., Yurdum L. M., Ozturk U., Isbilen E. & Cakmakoglu B. Association of the CC chemokine receptor 5 (CCR5) polymorphisms with preeclampsia in Turkish women. Archives of gynecology and obstetrics 286, 51–54, 10.1007/s00404-012-2244-3 (2012). 22314435

[b19] Du X. H. . SNP and mRNA expression for glutathione peroxidase 4 in Kashin-Beck disease. The British journal of nutrition 107, 164–169, 10.1017/S0007114511002704 (2012). 21733339

[b20] Practice A. C. o. O. ACOG practice bulletin. Diagnosis and management of preeclampsia and eclampsia. Number 33, January 2002. American College of Obstetricians and Gynecologists. International journal of gynaecology and obstetrics: the official organ of the International Federation of Gynaecology and Obstetrics 77, 67–75 (2002). 12094777

[b21] Alvarez-Fernandez I. . New biomarkers in diagnosis of early onset preeclampsia and imminent delivery prognosis. Clinical chemistry and laboratory medicine 52, 1159–1168, 10.1515/cclm-2013-0901 (2014). 24516004

[b22] Mol B. W. . Pre-eclampsia. Lancet 387, 999–1011, 10.1016/S0140-6736(15)00070-7 (2016). 26342729

[b23] Agarwal A., Aponte-Mellado A., Premkumar B. J., Shaman A. & Gupta S. The effects of oxidative stress on female reproduction: a review. Reproductive biology and endocrinology: RB&E 10, 49, 10.1186/1477-7827-10-49 (2012). 22748101 PMC3527168

[b24] Gohil J. T., Patel P. K. & Gupta P. Evaluation of oxidative stress and antioxidant defence in subjects of preeclampsia. Journal of obstetrics and gynaecology of India 61, 638–640, 10.1007/s13224-011-0094-8 (2011). 23204680 PMC3307937

[b25] Aumann K. D., Bedorf N., Brigelius-Flohe R., Schomburg D. & Flohe L. Glutathione peroxidase revisited–simulation of the catalytic cycle by computer-assisted molecular modelling. Biomedical and environmental sciences: BES 10, 136–155 (1997). 9315305

[b26] Sneddon A. A. . Regulation of selenoprotein GPx4 expression and activity in human endothelial cells by fatty acids, cytokines and antioxidants. Atherosclerosis 171, 57–65 (2003). 14642406 10.1016/j.atherosclerosis.2003.08.008

[b27] Crosley L. K. . The single-nucleotide polymorphism (GPX4c718t) in the glutathione peroxidase 4 gene influences endothelial cell function: interaction with selenium and fatty acids. Molecular nutrition & food research 57, 2185–2194, 10.1002/mnfr.201300216 (2013). 23934705 PMC4063342

[b28] Meplan C. . Functional effects of a common single-nucleotide polymorphism (GPX4c718t) in the glutathione peroxidase 4 gene: interaction with sex. The American journal of clinical nutrition 87, 1019–1027 (2008). 18400727 10.1093/ajcn/87.4.1019

[b29] Bermano G. . Evidence that a polymorphism within the 3′UTR of glutathione peroxidase 4 is functional and is associated with susceptibility to colorectal cancer. Genes & nutrition 2, 225–232, 10.1007/s12263-007-0052-3 (2007). 18850177 PMC2474949

[b30] Udler M. . Common germline genetic variation in antioxidant defense genes and survival after diagnosis of breast cancer. Journal of clinical oncology: official journal of the American Society of Clinical Oncology 25, 3015–3023, 10.1200/JCO.2006.10.0099 (2007). 17634480

[b31] Villette S. . A novel single nucleotide polymorphism in the 3′ untranslated region of human glutathione peroxidase 4 influences lipoxygenase metabolism. Blood cells, molecules & diseases 29, 174–178 (2002). 10.1006/bcmd.2002.0556 12490284

[b32] Maiorino M. . Genetic variations of gpx-4 and male infertility in humans. Biology of reproduction 68, 1134–1141, 10.1095/biolreprod.102.007500 (2003). 12606444

[b33] Lim J. H. . Genetic polymorphism of catechol-O-methyltransferase and cytochrome P450c17alpha in preeclampsia. Pharmacogenetics and genomics 20, 605–610, 10.1097/FPC.0b013e32833df033 (2010). 20729792

[b34] Ozturk E., Balat O., Pehlivan S., Ugur M. G. & Sever T. Genetic variation of myeloperoxidase gene contributes to preeclampsia: a preliminary association study in Turkish population. Hypertension in pregnancy 30, 377–383, 10.3109/10641955.2010.525278 (2011). 21827295

[b35] Procopciuc L. M. . The Ala-9Val (Mn-SOD) and Arg213Gly (EC-SOD) polymorphisms in the pathogenesis of preeclampsia in Romanian women: association with the severity and outcome of preeclampsia. The journal of maternal-fetal & neonatal medicine : the official journal of the European Association of Perinatal Medicine, the Federation of Asia and Oceania Perinatal Societies, the International Society of Perinatal Obstet 25, 895–900, 10.3109/14767058.2011.599078 (2012). 22432908

[b36] Khadzhieva M. B., Lutcenko N. N., Volodin I. V., Morozova K. V. & Salnikova L. E. Association of oxidative stress-related genes with idiopathic recurrent miscarriage. Free radical research 48, 534–541, 10.3109/10715762.2014.891735 (2014). 24499375

[b37] Polonikov A. V. . The C718T polymorphism in the 3′-untranslated region of glutathione peroxidase-4 gene is a predictor of cerebral stroke in patients with essential hypertension. Hypertension research : official journal of the Japanese Society of Hypertension 35, 507–512, 10.1038/hr.2011.213 (2012). 22158110

